# Enhancement of Transgene Expression by Mild Hypothermia Is Promoter Dependent in HEK293 Cells

**DOI:** 10.3390/life11090901

**Published:** 2021-08-30

**Authors:** Min Ho Jang, Honggi Min, Jae Seong Lee

**Affiliations:** Department of Molecular Science and Technology, Ajou University, Suwon 16499, Korea; minho00123@ajou.ac.kr (M.H.J.); minhk96@ajou.ac.kr (H.M.)

**Keywords:** CMV promoter, HEK293 cells, hypothermia, low culture temperature, transcription factor

## Abstract

Mild hypothermia has been widely used to enhance transgene expression and improve the cellular productivity of mammalian cells. This study investigated mild hypothermia-responsive exogenous promoters in human embryonic kidney 293 (HEK293) cells using site-specific integration of various promoter sequences, including CMV, EF1α, SV40, and TK promoters, into the well-known genomic safe harbor site, AAVS1. EGFP expression driven by the CMV promoter increased up to 1.5-fold at 32 °C versus 37 °C under stable expression, while others showed no hypothermic response. Integration of short CMV variants revealed that the CMV-enhancer region is responsible for the positive hypothermic response. CMV-enhancer-specific transcription factors (TFs) were then predicted through in silico analysis and RNA-sequencing analysis, resulting in the selection of one TF, *NKX3-1*. At 37 °C, overexpression of *NKX3-1* in recombinant HEK293 cells expressing EGFP through the CMV promoter (CMV-EGFP) increased EGFP expression up to 1.6-fold, compared with that in CMV-EGFP, the expression level of which was comparable to that of CMV-EGFP at 32 °C. Taken together, this work demonstrates promoter-dependent hypothermia responses in HEK293 cells and emphasizes interactions between endogenous TFs and promoter sequences.

## 1. Introduction

Human cell lines are a widely used mammalian expression host for the production of therapeutic glycoproteins [1]. Due to their ability to produce proteins including natural human products, the production of therapeutic proteins in human cell lines, particularly in human embryonic kidney 293 (HEK293) cells, is expanding [1,2]. HEK293 cells have been utilized as expression host cells for transient gene expression, but recent studies have established HEK293-based expression systems for stable and high-level production of therapeutic proteins [2,3]. To improve recombinant protein production, various approaches have been developed in industrially relevant mammalian cells such as CHO and HEK293 cell lines, among which expression under mild hypothermia (MH; 30–35 °C) is an effective method to extend culture longevity and improve productivity [4,5,6]. However, the beneficial effect of MH cultivation on increased productivity has been reported to be cell line specific [6,7,8]. Such clonal variation in transgene expression can originate from several factors [9], and recent studies have unveiled mechanistic insights into differences in protein expression and cell culture performance under MH, which include transgene integration sites, vector elements, regulation of specific genes involved in carbon metabolism and unfolded protein response, and expression of cold-shock proteins, albeit these studies are mainly conducted in CHO cells [8,10,11,12]. In the context of stable cell line development, the choice of a promoter is crucial because promoter activity determines both transgene expression levels and expression patterns under specific culture environments [8,9].

In this study, we evaluated the effect of widely used exogenous promoters on MH responses in HEK293 cells (specifically the HEK293E cell line). Stable expression levels of EGFP at 37 °C and 32 °C were directly compared using CRISPR/Cas9-mediated site-specific integration into the genomic safe harbor locus, adeno-associated virus site 1 (AAVS1) [13]. Given that interactions between transcription factors (TFs) and TF-binding sites (TFBSs) regulate transcription and alter gene expression levels [14], RNA-sequencing (RNA-seq) analysis was performed to identify TFs upregulated during MH. By comparing in silico analysis of TF and TFBS [15] in exogenous promoters and the significantly upregulated TFs from RNA-seq, we isolated one TF, *NKX3-1*, and tested the effect of *NKX3-1* overexpression on the increase in transgene expression. Low culture temperature enhanced transgene expression when driven by the CMV promoter, and CMV-enhancer specific TF overexpression improved transgene expression at 37 °C to a level similar to that observed in non-engineered cells at 32 °C. These data suggest that the MH response can be attributed to the interaction of exogenous promoters with endogenous transcription factors.

## 2. Materials and Methods

### 2.1. Cell Lines and Cell Culture

Adherent HEK293E cells from ATCC (ATCC number: CRL-10852) were maintained in Dulbecco’s modified Eagle’s medium (Gibco, Gaithersburg, MD, USA) supplemented with 10% fetal bovine serum (Hyclone, Logan, UT, USA) and 2 mM glutamine (Hyclone) and incubated at 37 °C in a humidified 5% CO_2_ atmosphere. The cells were grown in monolayer cultures in T flasks (Thermo Fisher Scientific, Waltham, MA, USA) with a working volume of 5 mL and passaged every 3 days. The HEK293E cell line containing a promoter-less EGFP expression cassette at the AAVS1 locus was generated in a previous study [13] and was maintained in culture media with 3 μg/mL puromycin (Sigma Aldrich, St. Louis, MO, USA). Viable cell density and viability were measured using an automated cell counter (Countess II FL, Invitrogen, Carlsbad, CA, USA).

### 2.2. Plasmids and Transfection

The sgRNA/Cas9 expression vector and donor plasmids for promoter knock-in (KI) were constructed as previously described [13]. Donor plasmids contained six different promoter sequences (EF1α, SV40, TK, CMV, CMV-core, or CMV-short) flanked by homology arms. All vector constructs were validated by Sanger sequencing and purified using NucleoBond Xtra Midi EF (Macherey-Nagel, Duren, Germany). Promoter KI pools were generated by transfecting the sgRNA/Cas9 expression vector and donor plasmid at a ratio of 1:1 (w/w) to the HEK293E cell line with promoter-less EGFP expression cassette using Lipofectamine 3000 (Invitrogen) according to the manufacturer’s instructions.

### 2.3. Flow Cytometry Analysis

At 48 h post-transfection, cells were divided into two different temperatures (37 °C and 32 °C) at densities of 0.2 × 10^6^ cells/mL. After 3 days of cultivation, the cells were resuspended in phosphate-buffered saline (PBS) supplemented with 10% FBS, and EGFP^+^ populations were measured using FACSCalibur (Becton Dickinson, Franklin Lakes, NJ, USA). The results were analyzed using FlowJo software (Tree Star, Ashland, OR, USA) to quantify the mean fluorescence intensity and percentage of EGFP^+^ populations.

### 2.4. RNA-Seq Analysis

Biological triplicate HEK293E samples, which were cultivated at 37 °C or 32 °C for 3 days, were used for RNA-seq analysis. Total RNA was isolated from approximately 2 × 10^6^ cells using TRIzol Reagent (Thermo Fisher Scientific, Waltham, MA, USA), and mRNA sequencing libraries were prepared using the Illumina TruSeq Stranded mRNA LT Sample Prep Kit (Illumina, San Diego, CA, USA) according to the manufacturer’s instructions. The samples were sequenced on an Illumina NovaSeq 6000 using reagents from the NovaSeq 6000 S4 Reagent Kit and 2 × 100 bp paired-end reads with approximately 70 million reads per sample. The raw data were trimmed with Trimmomatic 0.38 [16] to eliminate low-quality data and unnecessary artifacts. Reads were mapped to UCSC hg19 using HISAT2 (version 2.1.0) [17]. StringTie (version 1.3.4d) [18] was used for transcript assembly. With the read count value of the known gene obtained from transcript assembly, DEG analysis was performed using the DESeq2 R package [19]. Genes were considered DE if satisfying the condition |fc| ≥ 2 and nbinomWaldTest raw *p*-value < 0.05.

### 2.5. In Silico Analysis of TF and TFBS

Using JASPAR [15], the CMV-enhancer-specific TFs were identified under the condition of “relative profile score threshold = 80%”, which is the default setting of this system. The CMV promoter and CMV-short sequences were scanned to 810 human-specific TFs that were searched in the JASPAR database to identify putative TFBSs present in both sequences. Duplicate TFs were excluded to obtain the CMV promoter and CMV-short-specific TFs. CMV-enhancer-specific TFs were extracted by removing CMV-short TFs from the CMV-promoter TFs. Comparison of putative CMV-enhancer TFs and significant DEG lists from RNA-seq analysis resulted in 14 common TFs (Table S1).

### 2.6. Construction of NKX3-1 Overexpressing Cell Lines

To construct the *NKX3-1* expression vector, HEK293E cDNA was synthesized from isolated total RNA using the Maxima First Strand cDNA Synthesis Kit for RT-qPCR (Thermo Fisher). The *NKX3-1* cDNA fragment with *BamHI* and *XhoI* restriction sites was amplified from HEK293E cDNA using the following primers: forward primer: 5′-CTTGGATCCATGCTCAGGGTTCCGGA-3′, reverse primer: 5′-AGTCTCGAGTTACCAAAAAGCTGGGCTC-3′ (restriction sites are underlined) and then inserted into the pcDNA3.1/zeo (+) vector (Invitrogen). Stable cell pools overexpressing *NKX3-1* were constructed by transfecting the *NKX3-1* expression vector into the CMV-EGFP cell line [13] using Lipofectamine 3000, followed by the selection process using zeocin (200 μg/mL; Thermo Fisher). Control cells were prepared in the same manner as the pcDNA3.1/zeo (+) vector.

### 2.7. Quantitative Reverse Transcription PCR (RT-qPCR)

Total RNA isolation, cDNA synthesis, and measurement of relative mRNA expression levels were performed as previously described [20]. The primer sequences are listed in Table S2.

### 2.8. Statistical Analysis

Statistical significance was calculated using GraphPad Prism software (version 8.0.2; GraphPad Software, San Diego, CA, USA). Unpaired two-tailed *t*-tests were performed to determine the significance of differences. Data with *p* ≤ 0.05 were considered significant (* *p* ≤ 0.05, ** *p* ≤ 0.01, *** *p* ≤ 0.001).

## 3. Results and Discussion

**Figure 1:**
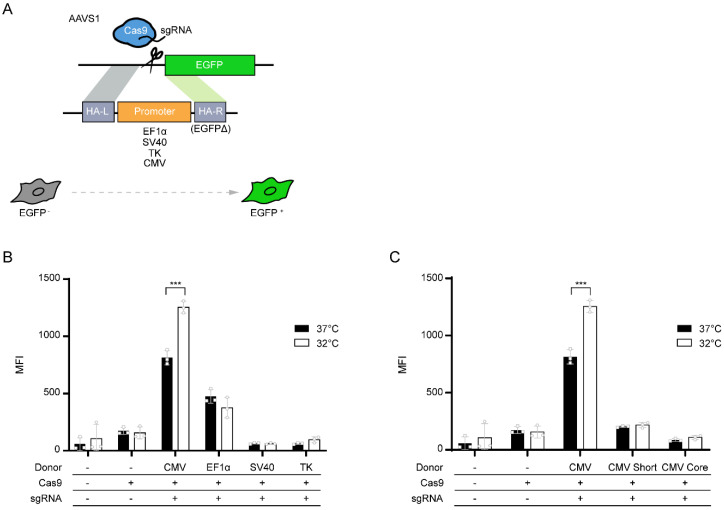
Promoter-dependent EGFP expression profiles of HEK293 cells at 37 °C and 32 °C: (**A**) schematic illustration of site-specific integration of four different promoters (EF1α, SV40, TK, and CMV) into the AAVS1 locus, in which a promoter-less EGFP expression cassette was integrated. CRISPR/Cas9-mediated targeted integration of promoters in each donor plasmid restored the expression of EGFP, enabling quantitative measurement using flow cytometry; (**B**) hypothermic cultivation of targeting pools. Expression levels of EGFP driven by each promoter were measured by flow cytometry, and the mean fluorescence intensity (MFI) of each sample is shown; (**C**) EGFP expression driven by either CMV promoter or short variants of CMV promoter, CMV-core, and CMV-short. Three promoters were integrated as shown in (**A**), and MFI was measured from targeting pools cultivated at 37 °C and 32 °C. In (**B**,**C**), each result of the experiment is depicted with dots. The error bars represent the mean ± standard deviation from three independent experiments. *** *p* ≤ 0.001 by two-tailed, unpaired *t*-test.

To identify which promoters are effective under MH conditions (32 °C) in HEK293 cells, we used the HEK293 cell line with a promoter-less EGFP expression cassette at the AAVS1 locus [13]. CRISPR/Cas9-mediated targeted integration of promoter sequences enables the restoration of EGFP expression, which can then be quantitatively analyzed by flow cytometry (Figure 1A). Transgene expression from identical integration sites can mitigate clonal variation typically shown in recombinant stable cell lines, enabling us to compare promoter dependence of transgene expression patterns under MH culture conditions. We integrated four commonly used exogenous promoters (CMV, EF1α, SV40, and TK) into HEK293 cells, followed by MH cultivation for 3 days. Flow cytometry analysis showed EGFP^+^ KI populations with different EGFP expression levels (Figure 1B and Figure S1). At 37 °C, the CMV promoter-driven EGFP expression was highest, followed by the EF1α, SV40, and TK promoters. Interestingly, the CMV promoter was the sole promoter showing increased EGFP expression up to 1.5-fold under MH, while other promoters showed no hypothermic response of EGFP expression (Figure 1B and Figure S1). This result is consistent with the promoter-dependent hypothermia response in CHO cells with increased transgene expression by the CMV promoter, while no difference in transgene expression was observed by the EF1α promoter under MH [8].

To further examine which specific regions of the CMV promoter contributed to this positive hypothermia response, we integrated two short variants of the CMV promoter, referred to as CMV-core and CMV-short, into the AAVS1 locus, and analyzed the hypothermia response (Figure 1C). CMV-core and CMV-short were short variants of the CMV promoter, indicating the core promoter region of the CMV promoter and the enhancer part-deleted CMV promoter, respectively (Figure S2). The two CMV-short variants significantly reduced the absolute EGFP expression level at 37 °C but did not respond to temperature shifts from 37 °C to 32 °C (Figure 1C). These results suggest that the CMV-enhancer region upstream of the CMV-core promoter could be involved in the positive hypothermic response.

**Figure 2:**
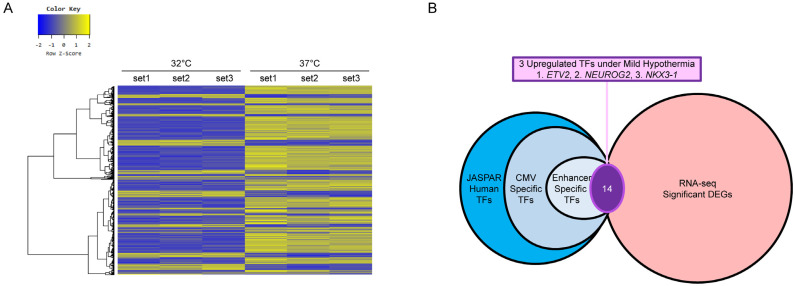
Identification of CMV-enhancer specific transcription factors (TFs): (**A**) heat map of the RNA-seq data. The color key represents the normalized value (log2 based) of the significant DEGs; (**B**) the schematic process of selecting TF candidates. The final three TFs were determined out of the common 14 results by comparing JASPAR and RNA-seq data.

Next, to identify TFs that were regulated in response to MH and may interact with the CMV region leading to increased EGFP expression, we performed comparative transcriptomic profiling (RNA-seq) of HEK293E cells under normal culture temperature (37 °C) and MH (32 °C) conditions. A total of 1517 genes were differentially expressed between 32 °C and 37 °C, among which 369 genes were upregulated and 1148 genes were downregulated (Figure 2A). Among the upregulated 369 differentially expressed genes (DEGs), in silico analysis identified three potential TFs, *ETV2*, *NEUROG2*, and *NKX3-1*, which were predicted to specifically bind the CMV-enhancer region (Figure 2B). RT-qPCR was performed to validate the upregulation of the three selected TFs upon MH treatment (Table S3). NKX3-1 showed comparable fold changes between RNA-seq (2.59-fold) and RT-qPCR (2.02-fold). However, *ETV2* and *NEUROG2* showed inconsistent results and lower fold changes in RT-qPCR (Table S3). This may be due to the lower sensitivity of RT-qPCR than of RNA-seq caused by low expression levels, which are supported by high threshold cycle values (>30) in RT-qPCR and low base mean (<150) in RNA-seq.

**Figure 3:**
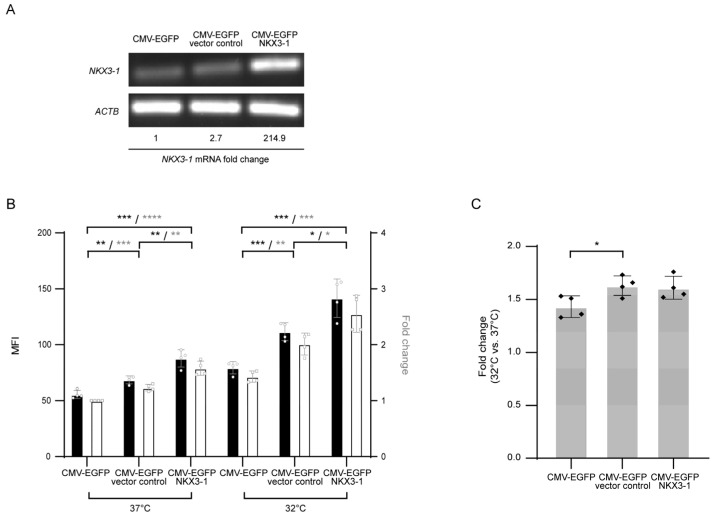
The effect of *NKX3-1* overexpression on mild hypothermia response: (**A**) RT-PCR analysis of CMV-EGFP derived cells with gene-specific primers. CMV-EGFP indicates EGFP expressing recombinant HEK293 cells. *NKX3-1*-overexpressing and empty vector control cells were established on the CMV-EGFP background. Fold change in *NKX3-1* mRNA expression relative to that in CMV-EGFP was assessed by RT-qPCR (Original gel images see Figure S3); (**B**) EGFP expression levels at 37 °C and 32 °C as measured by flow cytometry. The mean fluorescence intensity (MFI, black bars) and relative fold changes in MFI (white bars) are shown. Values are normalized to MFI of CMV-EGFP at 37 °C; (**C**) fold change in EGFP expression in each sample at 32 °C relative to expression at 37 °C. In (**B**) and (**C)**, the error bars represent the mean ± standard deviation from three independent experiments. * *p* ≤ 0.05, ** *p* ≤ 0.01, *** *p* ≤ 0.001, **** *p* ≤ 0.005 by two-tailed, unpaired *t*-test.

We then investigated the effect of overexpression of *NKX3-1*, a homeodomain-containing TF that plays a role as a putative prostate tumor suppressor [21], on CMV-driven EGFP expression in a recombinant HEK293 cell line expressing EGFP driven by the CMV promoter at the AAVS1 locus (CMV-EGFP) to test whether CMV-enhancer specific endogenous TFs (Figure S2) affect transgene expression patterns observed under MH conditions. *NKX3-1* overexpressing CMV-EGFP cells, together with the CMV-EGFP and *NKX3-1* empty vector control stable cell pools were cultivated at 37 °C and 32 °C, and EGFP expression levels were compared. *NKX3-1* was overexpressed more than 200-fold in the overexpression cell pool, compared to the empty vector control cell pools (Figure 3A). Interestingly, *NKX3-1* overexpression increased EGFP expression at 37 °C by 1.3 and 1.6 fold, compared to the *NKX3-1* control cell pools and CMV-EGFP, respectively (Figure 3B). Upon temperature shift from 37 °C to 32 °C, all cell lines showed enhanced EGFP expression up to 1.4 to 1.6 fold, which was not significantly different between cell lines; however, *NKX3-1* overexpressing CMV-EGFP cells showed a 2.6-fold increase in EGFP expression at 32 °C, compared to CMV-EGFP cells at 37 °C (Figure 3B). In addition, the EGFP expression level of *NKX3-1* overexpressing CMV-EGFP cells at 37 °C and CMV-EGFP cells at 32 °C had similar values (*p* > 0.05). This result suggests that *NKX3-1* overexpression can lead to an increase in transgene expression by the CMV promoter observed at 32 °C, even at 37 °C.

## 4. Conclusions

This study describes promoter-dependent responses to MH in HEK293 cells. The CMV-enhancer region may provide TFBSs for upregulated TFs under MH, leading to an increase in transgene expression. In addition to previous knowledge underlying the beneficial effects of MH, such as efficient metabolism and increased mRNA stability [22], we provide evidence supporting another mechanism: interactions between transgene regulatory elements and TFs. Based on this preliminary result, further studies could design various artificial promoters with TFBSs in the CMV-enhancer in combination with other types of promoters. Identification of the exact TFBS and optimal interaction of TFBS-TF and/or cofactor could facilitate the construction of industry-relevant MH inducible promoter-hybrids capable of both high basal transgene expression at 37 °C and higher activity under MH conditions than those achieved in natural promoters.

## Supplementary Materials

The following are available online at https://www.mdpi.com/article/10.3390/life11090901/s1, Figure S1: Representative flow cytometry plots and gates of CRISPR/Cas9-mediated targeted integration of promoter sequences at the AAVS1 locus shown in Figure 1B, Figure S2: Schematic representation of the short variants of the CMV promoter, Figure S3: Original gel images, Table S1: List of common transcription factors identified from comparison of in silico analysis of CMV-enhancer specific transcription factors and significant DEG lists from RNA-seq analysis, Table S2: Primer sequences used for RT-PCR and RT-qPCR, Table S3: Comparison of fold changes in DEGs identified by RNA-seq and RT-qPCR.
