# Supplementary material for: Enhancement of Transgene Expression by Mild Hypothermia Is Promoter Dependent in HEK293 Cells
Source: Life (Basel). 2021 Aug 30;11(9):901. doi: 10.3390/life11090901 (PMC8469586; doi:10.3390/life11090901)
Supplement: Supplementary file 1 [file life-11-00901-s001.zip › life-1340880-supplementary.pdf]

life

MDPI

Communication

Enhancement of Transgene Expression by Mild Hypothermia is Promoter-Dependent in HEK293 Cells

Min Ho Jang †, Honggi Min † and Jae Seong Lee *

Department of Molecular Science and Technology, Ajou University, Suwon 16499, Korea; minho00123@ajou.ac.kr (M.H.J.); minhk96@ajou.ac.kr (H.M.)
* Correspondence: jaeseonglee@ajou.ac.kr
† These authors contributed equally to this work.

Supplementary Materials

Table S1. List of common transcription factors identified from comparison of *in silico* analysis of CMV-enhancer specific transcription factors and significant DEG lists from RNA-seq analysis.

Gene Symbol	Gene Description	Fold Change (32 °C versus 37 °C)
FOS	Fos proto-oncogene, AP-1 transcription factor subunit	−3.86 (3.25×10^{-34})
RFX3	regulatory factor X3	−2.22 (8.52×10^{-6})
PRRX2	paired related homeobox 2	−2.03 (5.42×10^{-3})
FOSL1	FOS like 1, AP-1 transcription factor subunit	−4.51 (1.94×10^{-10})
LBX2	ladybird homeobox 2	−3.00 (3.23×10^{-6})
JDP2	Jun dimerization protein 2	−4.31 (1.80×10^{-22})
NEUROG2	neurogenin 2	2.17 (3.71×10^{-7})
EBF1	EBF transcription factor 1	−2.06 (1.97×10^{-3})
ETV1	ETS variant 1	−2.67 (8.10×10^{-8})
ETV2	ETS variant 2	2.61 (2.35×10^{-4})
NKX3-1	NK3 homeobox 1	2.59 (6.02×10^{-18})
ONECUT3	one cut homeobox 3	−3.08 (3.41×10^{-6})
HOXD9	homeobox D9	−2.25 (1.59×10^{-13})
INSM1	INSM transcriptional repressor 1	−3.03 (1.68×10^{-12})

Table S2. Primer sequences used for RT-PCR and RT-qPCR.

Gene	Direction	Sequence (5'-3')
ACTB	Forward	CTGGAACGGTGAAGGTGACA
	Reverse	AAGGGACTTCCTGTAACAACGCA
ETV2 isoform 1	Forward	AGCAGAGACATGCTGGAAAG
	Reverse	GGAACTTCTGGGTGCAGTAA
ETV2 isoform 2	Forward	CGCACGGACTGTACCATTT
	Reverse	GCTCTGGTACCGCTTCAAA
NEUROG2	Forward	CTGGGTCTGGTACACGATTG
	Reverse	CAGTCTACGGGTCTTCTTGATG
NKX3-1	Forward	CGGAGACCCAAGTGAAGATATG
	Reverse	CAAAGAGGAGTGCTTCTCCAA

Table S3. Comparison of fold changes in DEGs identified by RNA-seq and RT-qPCR.

Gene	Fold Change of DEGs (32 °C versus 37 °C)	
	RNA-seq (raw p-value)	RT-qPCR[a]
ETV2	2.61 (2.35×10^{-4})	1.36/1.12[b]
NEUROG2	2.17 (3.71×10^{-7})	1.24
NKX3-1	2.59 (6.02×10^{-18})	2.02

[a] Human *ACTB* gene was used as a reference gene. Average values obtained in triplicate experiments are shown. [b] *ETV2* isoform 1 / *ETV2* isoform 2.

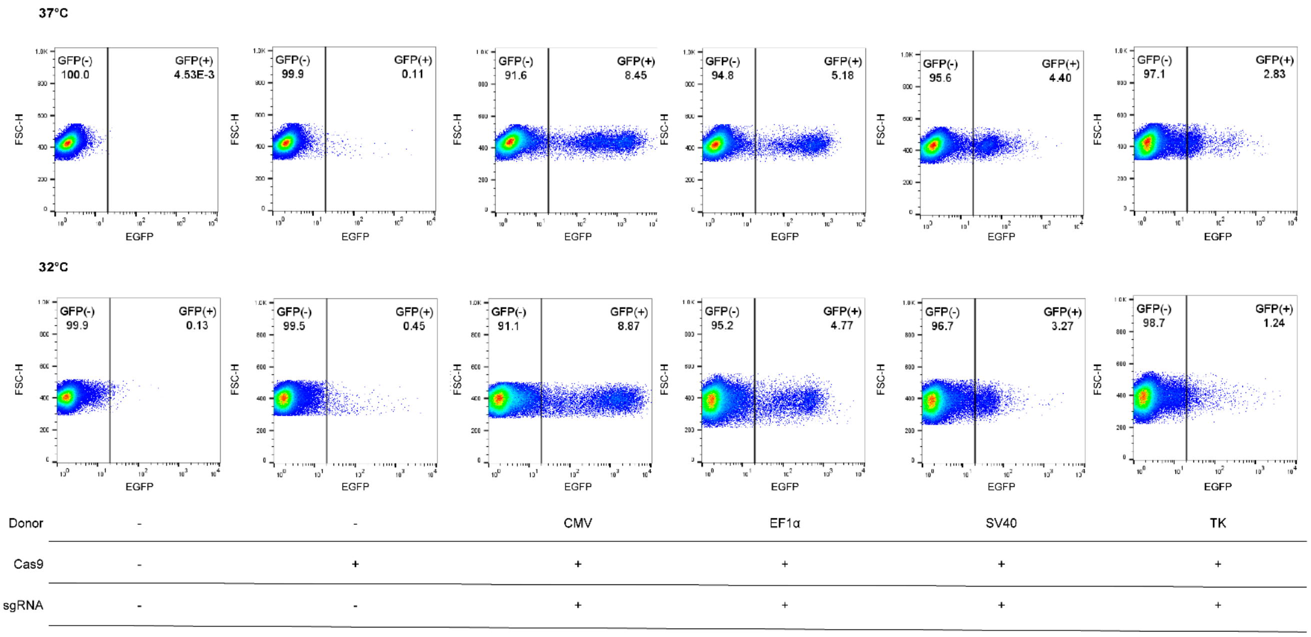

Figure S1. Representative flow cytometry plots and gates of CRISPR/Cas9-mediated targeted integration of promoter sequences at the AAVS1 locus shown in Figure 1B.

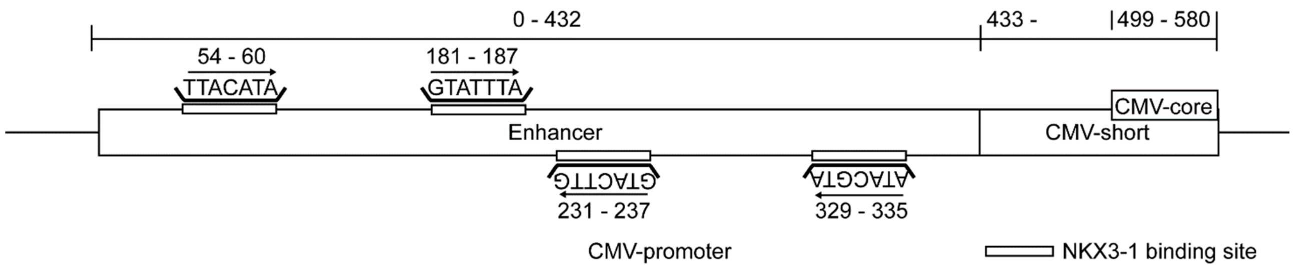

Figure S2. Schematic representation of the short variants of the CMV promoter. Predicted binding sites of NKX3-1 on the CMV-enhancer region are shown.

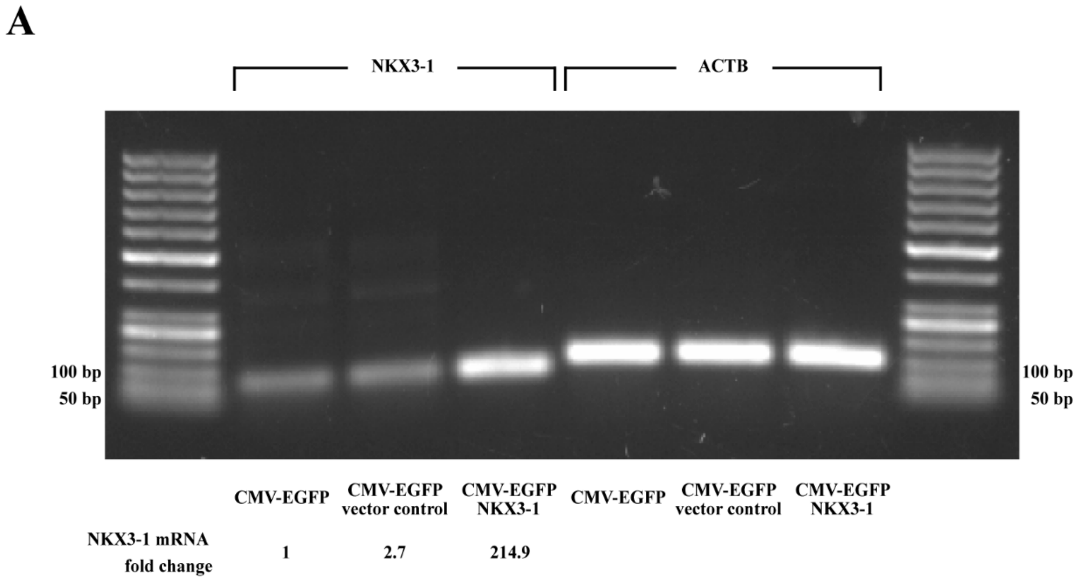

Figure S3. Original gel images.
